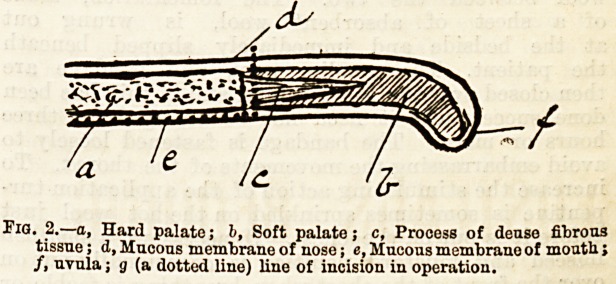# Treatment of Cleft Palate

**Published:** 1893-08-26

**Authors:** 


### THE HOSPITAL CLINIC.

[*The Editor will be glad to receive offers of co-operation and contributions from members of the profession. All letters should be addressed to* The Editor, The Lodge, Porchester Square, London, W.]

### ROYAL INFIRMARY, EDINBURGH.

### The Treatment of Cleft Palate.

In cases of well-marked cleft palate there are two serious inconveniences resulting from the absence of a complete septum between the cavity of the mouth and that of the nose. The first is the tendency which the food has to pass through the cleft and regurgitate through the nose, thus interfering with the nutrition of the patient as well as causing considerable discomfort. In many cases the patient learns to introduce the food in some particular way, and to hold the head in some such position as diminishes this tendency,

Fig. 1.—*a*, Foramen for anterior palatine artery; *b*, Posterior palatine artery; *c*, Position of sutures of relaxion; *d*, Edge of cleft pared; *e*, line of incision.

but in most it remains a source of trouble which can only be removed by surgical interference. In the second place, the interval in the palate interferes with the resonating function of the cavity of the nose, and gives to the voice a peculiar and characteristic nasal twang. In few cases is this improved by any means short of operation, and even then it is not entirely removed, because the soft palate cannot be so perfectly moulded into position as to completely shut off the communication between the two cavities in phonation, with the result that to a certain degree the nasal character persists even in the most favourable cases. It is with a view to mitigating these symptoms that the surgeon steps in in cases of cleft palate.

This developmental error is usually associated with some degree of hare-lip, sometimes on one side only, sometimes bilateral. In every case the notch in the lip should be closed up before the palate is touched, and this should be done as early as the surgeon can get permission to do it. The healing of the hare-lip facilitates the nourishment of the child by enabling it to suck; and it also exerts a beneficial influence on the cleft in the palate, which often shows a marked tendency to close after the lip has been put right, thus simplifying the operation which will have to be done at a later date to completely occlude it.

The question when to operate on cleft palate is not easily settled. If done at once the trouble from regurgitation of food is obviated, and the voice is more likely to be free of the nasal twang than if the operation is postponed for a year or two. On the other hand, the operation—at all times a difficult and delicate one—is especially so when the manipulations have to be carried out in the small mouth of a young infant. At a very early age children do not stand the loss of blood well, and the shock which follows on the operation is apt to prove serious. In addition, great difficulty is found in nourishing the child after the operation, as sucking is almost impossible, and the use of the spoon is not satisfactory. On the whole, it is best to cure the hare-lip at once, and leave the cleft in the palate till the patient is about two years old.

*Operation.*—The patient is thoroughly anæsthetised with chloroform, the mouth is kept open by an appropriate gag, and the edges of the cleft thoroughly pared with a sharp bistoury, the parts being kept tense by the aid of suitable forceps with which to grasp the posterior end of the palate. An incision (Fig. 1 *e*) is then carried from the level of the canine tooth in front to behind the last molar tooth, keeping as close to the teeth as possible. By taking care to go no further forward than the canine, and by working close to the alveolar margin, the anterior (*a*) and posterior (*b*) palatine arteries are left intact, and a broad area of tissue is left to keep up the vitality of the flaps to be raised. With a raspatory the whole of the soft tissues down to the bone are raised on each side, so that they can be pulled into contact in the middle line. They must be in perfect apposition before the stitches are introduced, otherwise the tension will inevitably prevent union.

There is often considerable difficulty in bringing the edges of the soft palate together partly on account of muscles, and partly because of a small process of dense fibrous tissue (Fig. 2 *c*) which passes into the soft palate from the posterior edge of the hard. Mr. John Duncan lays great stress on the importance of cutting everything that is in the least degree tight, and interfering with accurate and close approximation of the edges, and especially of carrying an incision through this fibrous prolongation and then through the nasal mucous membrane to ensure freedom. The edges are now brought accurately together by numerous stitches introduced with a cleft palate needle. The tension being greatest in the region where the hard and soft palates adjoin the stitches uniting the edges are apt to give way, leaving a small perforation in the new formed palate. To obviate this a deep stitch of fine silver wire should be inserted deeply into the substance of the palate to relax the tense structures (Fig. 1 *c*). Silver wire is used as being less irritating than silk, although it is somewhat more difficult to remove. The stitches are all removed at the end of a week or ten days, and

Fig. 2.—*a*, Hard palate; *b*, Soft palate; *c*, Process of dense fibrous tissue; *d*, Mucous membrane of nose; *e*, Mucous membrane of mouth; *f*, uvula; *g* (a dotted line) line of incision in operation.

during the first few weeks great care is taken in the feeding of the patient, so that no undue stretching of the palate takes place. The mouth is kept clean by frequent washing or sponging with boracic lotion, great gentleness being exercised lest coughing or retching be excited and the parts burst open.

In cases where only partial closure results from the operation, the patient is carefully nursed and fed up, and when all inflammation has subsided a second operation is performed on the same lines as the first.

## Figures and Tables

**Fig. 1. f1:**
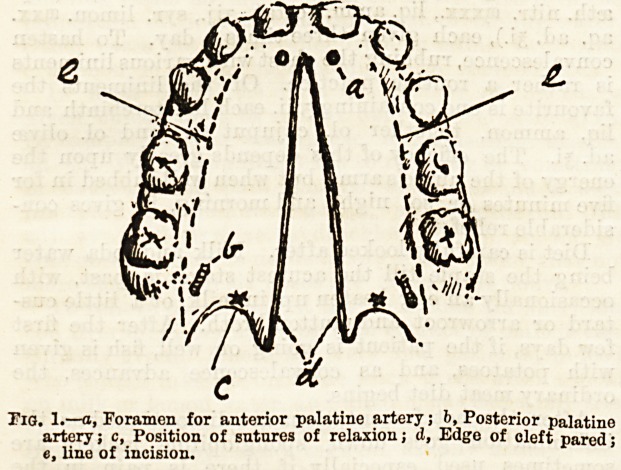


**Fig. 2. f2:**